# *Delftia* as a small-molecule chassis: lessons from delftibactin and harmane

**DOI:** 10.1128/aem.00277-25

**Published:** 2026-04-30

**Authors:** Pushkar Sai, Andrew Hoyek, Carlos C. Goller

**Affiliations:** 1 Department of Biological Sciences, North Carolina State University 6798 , Raleigh, North Carolina, USA; The Pennsylvania State University, University Park, Pennsylvania, USA

**Keywords:** *Delftia acidovorans*, *Delftia tsuruhatensis*, biocontrol, adaptation, bioremediation, small-molecule biosynthesis, comparative genomics, secondary metabolism, metallophores, harmane, delftibactin, small-molecule chassis

## Abstract

*Delftia* spp. occur in diverse environmental and host-associated settings, but their small-molecule capabilities are not uniform across the genus. In this Minireview, we use a strain-resolved “small-molecule chassis” framework to examine cases in which exported, low-molecular-weight metabolites can be linked to extracellular phenotypes. We focus on two anchor systems: delftibactin, a siderophore-like nonribosomal peptide metallophore associated with Au(III) detoxification and gold biomineralization in *Delftia acidovorans*, and harmane, a β-carboline linked to inhibition of early *Plasmodium* development by *Delftia tsuruhatensis* TC1 in mosquitoes. These systems do not represent the same level of mechanistic resolution, with delftibactin providing the tighter genotype→metabolite→phenotype chain and TC1 spanning both a harmane-linked mosquito phenotype and a less resolved low-molecular-weight supernatant phenotype in sand flies. Comparative genomics further supports the idea that these activities are strain- and lineage-specific rather than genus-wide. We argue that the small-molecule chassis framework is most useful not as a blanket label for *Delftia*, but as a way to identify which strains justify deeper mechanistic study and cautious translation.

## *DELFTIA* AS A SMALL-MOLECULE CHASSIS

*Delftia* spp. are widely distributed betaproteobacteria that have been identified across soils, plant-associated habitats, freshwater systems, and human-associated habitats (1–3). Phylogenomic analysis suggests that the genus is structured into two major lineages: an *acidovorans*-centered clade associated mainly with soil and rhizosphere environments, and a *lacustris*/*tsuruhatensis*-centered clade associated more often with sludge and human-associated settings (1). The same analysis also indicates a large accessory gene pool, consistent with substantial ecological and functional variation across strains (1). Here, rather than reviewing *Delftia* as a broadly versatile genus, we focus on what particular strains export: small molecules that measurably alter extracellular chemistry, microbial interactions, or host-associated phenotypes.

Rather than cataloging every reported trait of *Delftia*, we focus on cases where its ecological or host-associated effects are plausibly mediated by exported, low-molecular-weight metabolites. In this Minireview, we define “small-molecule chassis” as a strain that reproducibly occupies a relevant ecological or host-associated niche and generates measurable extracellular small-molecule activity that can be linked to a phenotype outside the cell. We use the term as a framework for comparing evidence across strains, not as a claim that all *Delftia* are equally suitable for application, particularly because some lineages are also represented among opportunistic clinical isolates. Importantly, this is not meant to imply uniform chemistry across the genus; instead, it provides a consistent way to compare evidence across strains and habitats, and to highlight where genotype-to-phenotype links remain unresolved (1).

Two case types best illustrate this framework. The first is an environmental chemistry case, where *D. acidovorans* uses delftibactin in metal-stressed settings. The second is a host-associated/vector case, where *D. tsuruhatensis* TC1 alters parasite outcomes in insects. In metal-stressed contexts, *D. acidovorans* produces delftibactin A, a nonribosomal peptide that chelates and transforms soluble, toxic Au(III) into inert gold nanoparticles, supporting survival during gold ion stress and contributing to gold biomineralization (4–6).

In vector biology, *D. tsuruhatensis* strain TC1 was reported to suppress malaria transmission by inhibiting early *Plasmodium* development via secretion of the β-carboline harmane (7). Importantly, related antiparasitic phenotypes appear to extend beyond harmane: in sand flies, TC1 disrupts *Leishmania* transmission, and its culture supernatant contains low-molecular weight factor(s) that impair *Leishmania* growth *in vitro*, yet harmane alone did not reproduce that effect in the same study, pointing to additional, undercharacterized chemistry and/or microbiome-mediated mechanisms (8, 9). These two anchor systems, therefore, illustrate different levels of mechanistic resolution: delftibactin is supported by a tighter metabolite-linked phenotype, whereas TC1-associated antiparasitic activity spans both a harmane-linked mosquito system and a less resolved low-molecular-weight supernatant phenotype in sand flies.

The goal of this Minireview is to synthesize what is currently supported by published literature about *Delftia* as a small-molecule chassis and organize the review around these two anchor systems. Specifically, we (i) briefly situate *Delftia* lineages and habitats as context for interpreting strain-level claims, (ii) summarize well-supported exported-metabolite examples (with delftibactin and TC1 as anchor cases), and (iii) highlight the major gaps that now limit translation, especially the need for clearer genotype→metabolite→phenotype linkages, better understanding of regulation and export, and prioritization of unresolved low-molecular-weight activities suggested by recent vector studies.

## ORIGINS OF *DELFTIA*

The genus name *Delftia* refers to the Dutch city of Delft, the site of isolation of the type species (10, 11). The species was previously classified in the genus *Comamonas* before the name *Delftia* was formally proposed in 1999 following a taxonomic re-evaluation (10). Over time, six type species have been described within the genus and were named as follows: *D. acidovorans* in 1999 (10)*, D. tsuruhatensis* in 2003 (12)*, D. lacustris* in 2009 (3), *D. litopenaei* in 2012 (13), *D. deserti* in 2015 (14), and *D. rhizosphaerae* in 2017 (15), with isolates reported from soil, water, sludges, and human-associated settings (3, 16). Members of the genus *Delftia* exhibit certain morphological features, including a gram-negative, rod-shaped, non-spore-forming cell structure, alongside characteristic physiological traits, such as non-fermentative metabolism and nitrate reduction (10, 17, 18). Single cells typically measure between 0.4 and 0.8 µm in width and 2.5 and 4.1 µm in length (10). Over the years, these organisms have gained increased scientific attention due to their remarkable metabolic flexibility and ecological adaptability (1, 17–21). Nonetheless, the genus’ environmental, metabolic, and potential pathogenic traits remain poorly understood (22–24).

### Assessing the “small-molecule chassis” viewpoint in the wild

**Fig 1:**
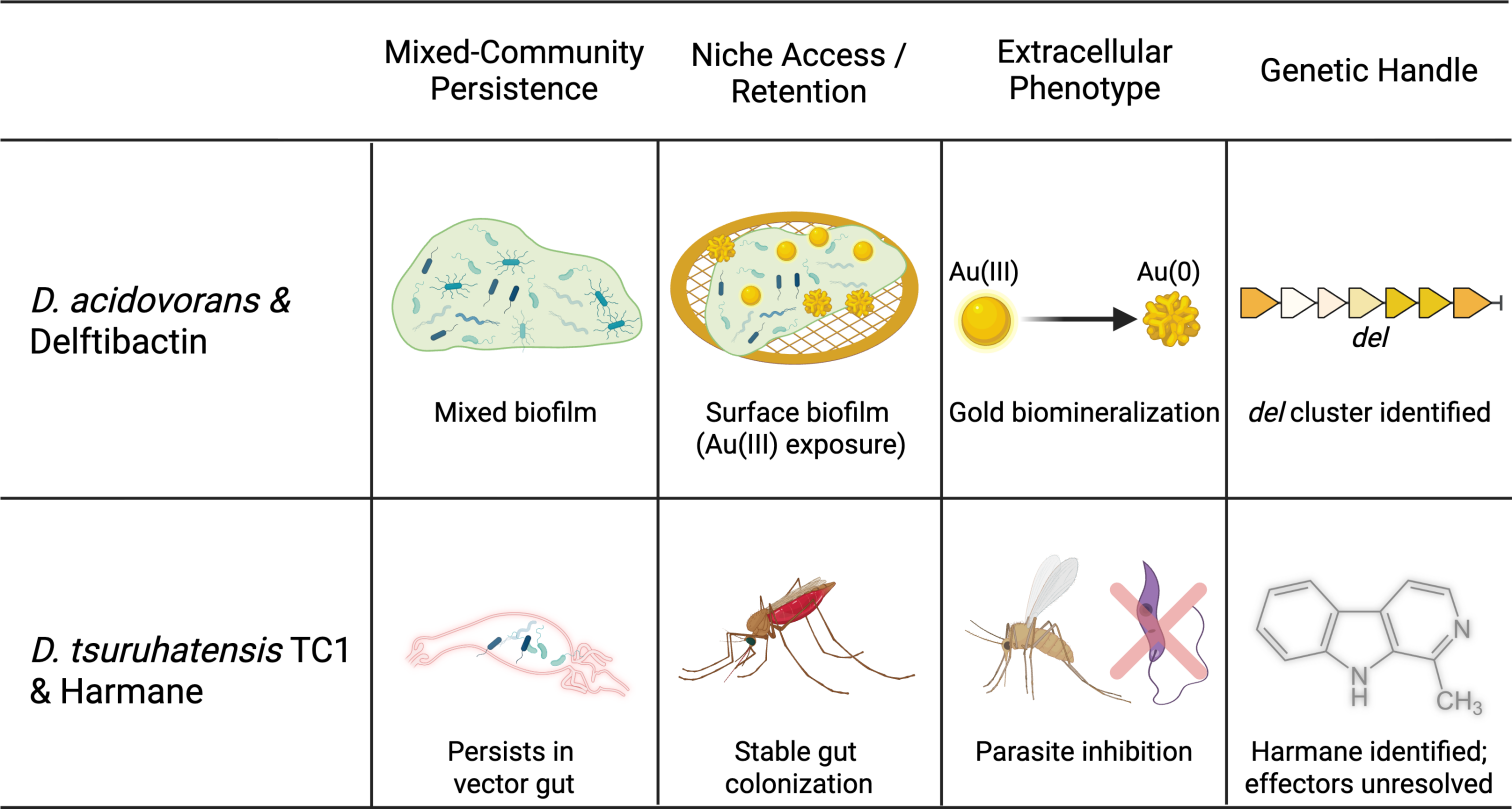
Operational criteria for evaluating *Delftia* as a "small-molecule chassis." A four-part framework—community persistence, niche access/retention, extracellular phenotype, and the genetic basis—is applied to two anchor systems. In *D. acidovorans*, mixed-community persistence is supported by coaggregation and multispecies biofilm formation (1, 25); niche access/retention by biofilm-associated survival under Au(III) exposure (5); extracellular phenotype by delftibactin-linked Au(III) detoxification and biomineralization (6); and genetic handle by identification of the *del* biosynthetic cluster (1, 6). In *D. tsuruhatensis* TC1, mixed-community persistence and niche access/retention are supported by stable colonization of the mosquito/sandfly gut (7, 8, 26), extracellular phenotype by inhibition of early *Plasmodium* development, with harmane identified as one secreted active metabolite (7), and by low-molecular-weight activity that disrupts *Leishmania* transmission but is not fully explained by harmane alone (8, 9); and genetic handle remains unresolved beyond identification of harmane as one active molecule (7, 8, 27). *“Delftia* Anchor Systems” by P. Sai created in BioRender (https://BioRender.com/avbpp6a) is licensed under CC BY 4.0.

Treating *Delftia* as a “small-molecule chassis” is only useful if the concept can be tested in real ecological settings. Here, we evaluate the idea using four criteria: (i) persists in mixed communities, (ii) occupies the relevant niche, (iii) produces measurable extracellular activity, and (iv) has sufficient genetic evidence to connect strain, metabolite, and phenotype. In practice, this means combining comparative genomics with experiments that measure niche colonization and extracellular activity in culture supernatants or fractions. We use this checklist throughout the Minireview and summarize how it maps onto the two anchor examples discussed here, delftibactin and TC1, in Fig. 1.

### Persistence in mixed communities

A useful chassis must function in mixed communities, where interspecies interactions shape persistence, competition, stress tolerance, and cross-feeding. At the genomic scale, *Delftia* shows substantial strain-level diversity. A genus-wide phylogenomic and pangenome analysis of 61 genomes reported an open pangenome (>28,000 genes) and a relatively small core genome (884 genes), supporting the idea that specialized functions, including small-molecule biosynthetic capacity, are unevenly distributed across strains (1). The same analysis resolved two major lineages with distinct habitat associations: an *acidovorans*-centered clade enriched for soil/rhizosphere isolates, and a *lacustris*/*tsuruhatensis*-centered clade enriched for sludge/human-associated isolates (1). This further argues against treating metabolite-linked capacity as genus-universal. In practice, this means chassis claims should be tested by asking whether lineage- or strain-specific accessory loci track with extracellular metabolite profiles and community-visible phenotypes. By community-visible phenotype, we mean a measurable effect that a *Delftia* strain exerts on the structure, behavior, or function of a surrounding microbial community, even when that effect is not attributable to a defined exported small molecule. At the community level, a drinking-water isolate of *D. acidovorans* coaggregated with partner bacteria and facilitated multispecies biofilm development, consistent with persistence in mixed communities (28). In host-associated settings, TC1 also persists within the naturally mixed midgut community across mosquito life stages, supporting the idea that its effects can persist under non-axenic conditions (7). Within this framework, persistence in mixed communities matters because it increases the likelihood that extracellular activities will be produced, encountered, and remain ecologically relevant under realistic conditions.

### Access to the relevant physical niche

Exported metabolites matter only if producing cells reach and remain in the place where those molecules are expected to act; niche access and retention are, therefore, core chassis features. In plant-associated contexts, *D. acidovorans* RAY209 attaches to roots during early host interaction, and transcriptomic profiling captured a shift from a suspended state to a root-attached lifestyle during colonization (28). This supports the idea that any secreted chemistry can be delivered at the plant interface rather than inferred only from planktonic culture. In vector biology, *D. tsuruhatensis* strain TC1 colonizes the mosquito midgut across life stages, a practical prerequisite for any claim that a secreted metabolite can act *in vivo* (7). Traits, such as motility, may help cells access these niches, but they are relevant here mainly insofar, as they make secreted chemistry deliverable *in situ* (3, 13–15, 29). Within this framework, physical colonization traits matter because they are interpreted primarily as *delivery infrastructure* for secreted chemistry, not as the central phenomenon.

### Extracellular activity with clear, measurable phenotypes

The “small-molecule chassis” framework is strongest when a defined metabolite can be linked to a measurable extracellular phenotype. By extracellular phenotype, we mean a measurable effect expressed outside the bacterial cell—for example, a change in extracellular chemistry, biomineralization, or host-associated biology—that can be linked to secreted or cell-released small molecules. Delftibactin is the clearest current example: it was first linked to gold detoxification and biomineralization in *Delftia* (6, 30), and later work strengthened that case through structural/mechanistic characterization and biofilm-based experiments under Au(III) exposure (4, 5). A second anchor comes from vector biology: *D. tsuruhatensis* TC1 suppresses malaria transmission, with harmane identified as a secreted small-molecule inhibitor of early *Plasmodium* development in the mosquito midgut (7). Recent work in sand flies extends the story but also makes it more complex. TC1 can disrupt *Leishmania* transmission, and bacterial supernatant fractions contain low-molecular-weight activity. However, harmane alone does not account for the full phenotype in that system (8). Together, these studies show that *Delftia* can produce extracellular small-molecule phenotypes across very different settings, but they do not provide the same level of mechanistic resolution. Delftibactin remains the tighter metabolite-linked case, whereas TC1 currently spans both a harmane-linked mosquito phenotype and a less resolved low-molecular-weight supernatant phenotype in sand flies (8).

### Genetic basis: linking genotype→metabolite→phenotype

A chassis concept becomes substantially more useful when an extracellular phenotype can be tied to identifiable genes and compared across strains. For delftibactin, the early discovery work (6) and recent structural/mechanistic characterization (4) provide a concrete molecular anchor. Genus-level phylogenomics further shows that the *del* biosynthetic cluster is present in many *Delftia* genomes but incomplete or absent in subsets of strains, reinforcing that this metabolite capacity is lineage- and strain-structured rather than genus-universal (1, 4, 6). By contrast, TC1-associated antiparasitic phenotypes remain genetically underresolved. Closing that gap will require linking strain-resolved loci to metabolite production and vector-associated phenotypes across comparable backgrounds (7, 8). Accordingly, statements that “*Delftia* produces X” should be treated as strain-level claims supported by strain-resolved genotype evidence and interpreted in phylogenomic context.

## DELFTIBACTIN-MEDIATED GOLD BIOMINERALIZATION

Delftibactin is one of the clearest *Delftia* cases for the small-molecule chassis framework because it links its metabolite to a measurable extracellular phenotype. As such, *D. acidovorans* mitigates Au(III) stress by producing delftibactin, which promotes conversion of toxic dissolved gold into inert extracellular gold particles (6).

### Phenotype and chassis behavior

Johnston et al. (6) originally described delftibactin as a metallophore linked to gold biomineralization in *D. acidovorans* and framed protection from soluble gold as a metabolite-mediated process (i.e., the “work” is done by exported chemistry interacting with an abiotic stressor) (6). Importantly, the phenomenon is not restricted to bulk culture.

Funari et al. (5) examined gold biomineralization in a surface-associated context by growing *D. acidovorans* biofilms on gold-coated quartz and tracking responses during Au(III) exposure using quartz crystal microbalance measurements (5). This is important because it shows that delftibactin-associated chemistry can operate in biofilms, where local concentration, diffusion, and surface effects make extracellular metabolites especially consequential (5, 31).

More recently, Takeuchi et al. (4) determined the full structure of delftibactin A and linked it to reductive gold nanoparticle formation, further strengthening the genotype→metabolite→phenotype relationship (4). Altogether, these studies make delftibactin the most mechanistically resolved example in this review.

The functional picture of delftibactin has also broadened beyond gold detoxification alone. Tejman-Yarden et al. (32) reported that purified delftibactin A showed antimicrobial activity against several clinically relevant bacteria and interpreted the compound as a siderophore-like metallophore with possible roles in metal acquisition and transport. Their experiments further suggest that delftibactin can participate in metal-handling processes beyond Au(III), although the relative ecological importance of these functions in native *Delftia* settings remains unresolved (32). This broader view is useful because it frames delftibactin as a multifunctional extracellular metal-binding metabolite rather than only as a gold-specific case.

Lastly, delftibactin-like chemistry also extends beyond a single strain background. Liquid chromatography-mass spectrometry (LC-MS) metabolomics and genome mining in *D. lacustris* DSM 21246 identified lipophilic delftibactin analogs (delftibactins C–F) induced under iron limitation, indicating that the scaffold can vary across strains and respond to ecologically familiar metal-limitation conditions (33). Altogether, delftibactin remains the strongest current example of a *Delftia* small-molecule chassis: a strain-resolved system in which a defined exported metabolite is linked to a clear extracellular phenotype, supported by structural characterization, ecological context, and emerging evidence for broader metal-related and antimicrobial functions.

## HARMANE-MEDIATED ANTIPARASITIC ACTIVITY

### TC1 suppresses *Plasmodium* in *anopheles*

The TC1 story provides a strong example that a *Delftia* strain can function as a small-molecule chassis in a vector-relevant niche. Huang et al. (7) isolated *Delftia tsuruhatensis* TC1 from mosquitoes incapable of sustaining *Plasmodium falciparum* development. The study showed that TC1 inhibits early parasite development in the mosquito midgut and reduces transmission potential (7). Importantly, the phenotype is associated with exported chemistry: harmane was identified as the secreted inhibitor responsible for the anti-*Plasmodium* effect (7).

Two features make this one of the clearest *in vivo* small-molecule cases in the review. First, TC1 stably populated the mosquito gut and inhibited *Plasmodium* development across the mosquito’s life, satisfying the basic requirement that cells persist in the right place long enough for secreted chemistry to matter (7). Second, harmane was shown to penetrate the mosquito cuticle upon contact and still inhibit parasite development, supporting the idea that the phenotype can be mediated by the molecule itself rather than requiring direct bacterial–parasite contact (7). Collectively, these findings support a relatively direct model in which a secreted effector mediates activity in the mosquito–*Plasmodium* system.

### TC1 disrupts leishmaniasis transmission

A key mechanistic uncertainty emerges when TC1 is moved into a different vector/parasite system. Cecílio et al. (8) showed that TC1 colonizes the midgut of *Phlebotomus duboscqi* sand flies and reduces *Leishmania major* development, lowering transmission to mice (8). Their data suggest the phenotype is not indirect, such as through TC1-driven gut dysbiosis, and cannot be explained solely by a single identified metabolite (8).

The key question is whether secreted products contribute directly to this effect. Cecílio et al. found that bacteria-free TC1 culture supernatant impaired *L. major* growth *in vitro* at sufficiently high supernatant fractions, and that the activity localized to components smaller than 10 kDa; however, harmane alone did not reproduce the inhibition in their assays (8).

This points to a narrower and more defensible conclusion: TC1-associated antiparasitic activity can involve low-molecular-weight extracellular factors beyond harmane, and the dominant mechanism may differ across vector contexts. In mosquitoes, the data fit a relatively direct harmane-linked model; in sand flies, the current evidence is more consistent with a mixed system involving unresolved low-molecular-weight activity and microbiome-mediated effects (7, 8).

This interpretation is also consistent with the broader β-carboline literature: harmane and related β-carbolines have reported antileishmanial activity *in vitro* (9), but these observations do not establish that harmane is the relevant effector in the TC1 sand fly system when harmane fails to reproduce TC1-associated effects under the tested conditions (8, 9). Together, the mosquito and sand fly studies frame TC1 as a credible vector-colonizing small-molecule chassis with reproducible antiparasitic phenotypes while defining a tractable next step for the field: identify the <10 kDa factor(s) involved in the sand fly system and separate direct secreted-molecule effects from microbiome-mediated mechanisms.

## EMERGING APPLICATIONS OF THE SMALL-MOLECULE CHASSIS

The two anchor case studies provide the clearest examples of small-molecule chassis behavior: the strain occupies the relevant niche; extracellular activity is detectable; and the phenotype is expressed outside the cell. The practical question is how broadly that logic should be applied. The strongest application cases are those in which a *Delftia* strain produces an external chemical change *in situ*, while broader metabolic capabilities and production-host uses are better regarded as provisional extensions of the framework. That caution matters because *Delftia* includes lineages associated with opportunistic infection and should not be treated as a uniformly deployable genus.

Environmental applications are currently the most straightforward because success can be defined using external, measurable endpoints, such as metal speciation, precipitation, contaminant disappearance, or product formation, without requiring interpretation through host biology. Several *Delftia* reports fit that framing. In heavy-metal contexts, *D. acidovorans* strain Pb11 has been reported to reduce Pb(II) to elemental Pb(0) (metallic lead), with growth under Pb(II) exposure and visible Pb(0) precipitation under the tested conditions (34). Similarly, *Delftia* sp. JD2 has been shown to reduce Cr(VI) to Cr(III) via an NAD(P)H-dependent reductase and has been shown to retain plant growth-promoting traits in the same study context (35) useful because it displays coupling of an “external detox” to a plausible deployment niche in contaminated soils. Field-adjacent work also exists: inoculation with cadmium-resistant *Delftia* sp. B9 reduced Cd accumulation in rice grains grown in Cd-contaminated soil (36). Together, these studies suggest that some *Delftia* strains can mediate environmentally relevant metal-fate changes in settings that are at least adjacent to deployment.

Pollutant degradation is a less direct fit to the small-molecule chassis framework because the measurable outcome is usually substrate turnover rather than a defined exported effector. Even so, *Delftia* repeatedly appears in degradative settings where community-visible chemistry can be measured cleanly, including degradation of phenols, phenanthrene, 2,4-D, dimethylphenol isomers, and acetaminophen (37–41). The implication is not that *Delftia* is uniquely capable, but that some strains repeatedly occur in systems where external chemical change can be followed with straightforward outcome metrics. These cases are therefore better viewed as broader community-visible metabolic transformations than as direct exported-metabolite applications.

Bioproduct formation is an even looser extension of the framework. Engineering *D. acidovorans* DSM39 to produce polyhydroxyalkanoates (PHAs) from slaughterhouse waste shows that some strains can function as useful production hosts, but this is better interpreted as a strain- and pathway-specific host-platform example than as direct evidence of exported small-molecule chassis behavior (42).

Vector deployment is promising, but it also imposes the highest evidentiary bar because efficacy and safety must both be demonstrated *in situ*. The mosquito and sand fly studies provide the basic minimum evidence for a TC1 chassis claim: stable colonization, a reproducible antiparasitic outcome, and at least partial linkage to low-molecular-weight effectors or supernatant activity (7, 8). At the same time, the literature justifies a clear caution. *Delftia* includes opportunistic pathogens, and clinical isolates and infections have been documented for both *D. tsuruhatensis* and *D. acidovorans* (17, 24, 43). In practice, no vector deployment claim will be persuasive without evidence that both efficacy and safety hold under realistic field conditions.

## CONCLUSION

Together, these case studies support a strain-resolved view of *Delftia* as a small-molecule chassis, but only when the term is applied with clear evidentiary limits. Delftibactin remains the strongest current example: a defined exported metabolite linked to a measurable extracellular phenotype and supported by a relatively tight genotype→metabolite→phenotype chain. TC1 provides a second, biologically compelling case in vector-associated settings, but its antiparasitic activity is not yet resolved to the same extent across host contexts, particularly in the sand fly system.

These differences matter because the relevant capabilities are not genus-universal. Instead, they are distributed unevenly across strains and lineages, reinforcing the need for strain-resolved links among genes, metabolites, and phenotypes. The major gaps now are, therefore, clear: better definition of biosynthetic loci, stronger understanding of regulation and export, and systematic identification of unresolved low-molecular-weight activities suggested by recent vector studies.

In practical terms, the most direct near-term applications are those in which *Delftia* alters an external chemical state that can be clearly measured, such as in metal-fate transformations. Other applications, including pollutant degradation, bioproduct formation, and vector deployment, should be treated more cautiously and evaluated case by case, especially given the opportunistic clinical relevance of some *Delftia* lineages. The value of the small-molecule chassis framework is, therefore not that it makes all *Delftia* deployable, but that it helps identify which strains justify deeper mechanistic study and which, if any, merit translation.
